# Assessing the needs and perspectives of patients with obesity and obstructive sleep apnea syndrome following continuous positive airway pressure therapy to inform health care practice: A focus group study

**DOI:** 10.3389/fpsyg.2022.947346

**Published:** 2022-09-09

**Authors:** Giada Rapelli, Giada Pietrabissa, Licia Angeli, Ilaria Bastoni, Ilaria Tovaglieri, Paolo Fanari, Gianluca Castelnuovo

**Affiliations:** ^1^Psychology Research Laboratory, Istituto Auxologico Italiano IRCCS, Milan, Italy; ^2^Department of Psychology, Catholic University of the Sacred Heart, Milan, Italy; ^3^Pulmonary Rehabilitation Department, Istituto Auxologico Italiano IRCCS, Verbania, Italy

**Keywords:** obstructive sleep apnea syndrome, continuous positive airway pressure, obesity, focus group, interpretative phenomenological analysis, clinical psychology

## Abstract

**Objective:**

This study aims to investigate the lived experience in patients with obstructive sleep apnea syndrome (OSAS) and comorbid obesity following after continuous positive airway pressure (CPAP) therapy made with the disease the device, and to identify barriers and facilitators to the use of CPAP to improve rehabilitation provision and aid in disease self-management.

**Methods:**

Qualitative research was conducted using three focus groups with a representative sample of 32 inpatients (37% female) undergoing a 1-month pulmonary rehabilitation program at the IRCSS Istituto Auxologico Italiano San Giuseppe Hospital, Verbania, Italy. The focus groups were recorded on tape, and contemporaneous notes were made. The tapes were transcribed verbatim, and Interpretative Phenomenological Analysis was used to develop themes.

**Results:**

Six main themes were extracted: (1) Living the diagnosis as a shock; (2) You should not sleep on it: the importance of prevention; (3) The adjustment to CPAP; (4) Barriers and facilitators to the use of CPAP; (5) Three in a bed; and (6) The relationship with the healthcare system.

**Conclusion:**

Results of this study suggest potential avenues for interventions to increase adherence to CPAP, including the provision of information and continued support. Individual counseling providing strategies aimed at helping the person to cope with the emotional problem and relational difficulties associated with the use of CPAP, and at strengthening self-efficacy and self-management skills are also encouraged for optimal care during the rehabilitation program.

## Introduction

Obstructive sleep apnea syndrome (OSAS) is an increasingly prevalent chronic sleep disorder affecting 3–7% of adult men and 2–5% of adult women worldwide (Lyons et al., 2020). It is characterized by a partial or complete obstruction of the upper airway affecting sleep quality and ensuing daytime fatigue and sleepiness (Cancino and Rivera, 2018; Gibson et al., 2018).

Obstructive sleep apnea syndrome is frequently associated with several clinical problems, including hypertension, cardiovascular disease, abnormal glucose metabolism, and reduced quality of life (Garvey et al., 2015).

Additionally, studies show that patients with OSAS utilize health care resources almost twice as much as control patients do (Lee et al., 2008).

Weight gain is commonly known to be the most important risk factor for developing OSAS. Furthermore, there is a bidirectional relationship between obesity and OSAS, with OSAS potentially leading to faster weight gain, and the entertainment of a vicious cycle of increasing weight and worsening OSAS (Ong et al., 2013).

Continuous positive airway pressure (CPAP) is the main treatment for OSAS symptoms. It has been shown to reduce breathing disturbances, increase blood pressure, and decrease mortality rates, and was associated with improved daytime sleepiness, and daily functioning in several investigations (Ye et al., 2017; Adams et al., 2020).

However, the success of this treatment relies on treatment adherence, which is frequently suboptimal in this population of patients (Weaver and Grunstein, 2008; Rapelli et al., 2021a; Gabryelska et al., 2022).

Improving CPAP adherence is commonly a challenging process—as affected by different factors including age, gender, symptoms severity, socioeconomic and marital status, perceived social support, and technical issues associated with the device (Mehrtash et al., 2019). The acceptance of CPAP is a process that is perceived as a difficult challenge and requires a period of adjustment that is frequently associated with the personal perception and experience of patients (Gibson et al., 2018; Rezaie et al., 2021). In fact, patients often seek an alternative before accepting CPAP, and even when they decide to adhere to CPAP, they may do it with skepticism (Rezaie et al., 2021). This period is often associated with negative perceptions of CPAP and this may hinder acceptance (Gibson et al., 2018). Another key concern for patients regards their appearance and body image while wearing the mask in front of other people because they feel embarrassed and uncomfortable (Luyster et al., 2016; Shapiro and McCrone, 2017; Ward et al., 2017; Ye et al., 2017). Also, CPAP is often described as bothersome during the night because of the difficulties to adjust the mask, the noises that disturb sleep, the air directed toward the bed partner, and the consequent loss of intimacy with the partner (Luyster et al., 2016; Ye et al., 2017). CPAP may also represent a problem for traveling patients: from a logistic viewpoint, the device is difficulty difficult to carry and it needs electricity to function (Luyster et al., 2016).

Pulmonary rehabilitation is effective in reducing symptoms among patients with OSAS, providing disease education and self-management components (Ward et al., 2002; Pearson, 2004; Nici et al., 2006) to improve exercise capacity, quality of life, and the patient’s understanding of the disease and treatment (McCarthy et al., 2015).

However, a few studies examine the understanding or information needs of patients with OSAS in CPAP, or whether the educational component of pulmonary rehabilitation meets these needs.

Examining the perspectives and lived experiences of this population is fundamental to gaining a better understanding of their needs, and factors that facilitate and hinder CPAP use.

To this aim, the present study employs a qualitative method using focus groups, (1) to assess knowledge, beliefs, and attitudes about OSAS syndrome and CPAP therapy, and (2) to identify specific barriers and facilitators to CPAP use among patients with OSAS and comorbid obesity.

The results presented in this article are from the preliminary phase of a larger project to develop and implement a telephone-based behavioral and motivational intervention to improve adherence to recommended OSAS treatment (Rapelli et al., 2022).

By identifying patients’ needs, researchers and health professionals may be able to develop effective interventions capable of reducing the sleep health disparities observed in this population.

## Materials and methods

A qualitative research design using focus groups was used to elicit an in-depth understanding of the participants’ perceptions about OSAS and to identify barriers preventing the utilization of CPAP. This methodological approach builds on group interaction and is especially valuable for capturing how views are constructed and negotiated (Kitzinger and Barbour, 1999; Dahlgren et al., 2007).

The study was approved by the Ethics Committee of the IRCCS Istituto Auxologico Italiano, Milan, Italy (research project code: 2021_03_23_02; acronym: MotivAir) and was in accordance with the Helsinki Declaration of 1975, as revised in 2008. The purpose and objective of the study were explained to each subject and written informed consent was obtained before starting the study.

### Participants

Participants were recruited from the Department of Pulmonary Rehabilitation of the IRCCS Istituto Auxologico Italiano, San Giuseppe Hospital, Verbania, Italy.

They were selected for inclusion in the study during their first week of the pulmonary and metabolic rehabilitation program, according to the following criteria: (1) having a diagnosis of OSAS confirmed with polysomnography;(2) being recommended for the use of CPAP for at least 6 months; (3) having a body mass index, BMI ≥ 30 kg/m^2^; (4) being 18 years or older; and (5) being Italian speakers. Subjects with severe apnea, and cognitive or hearing impairments were excluded from the study. Before enrollment in the study, all patients were informed in detail about the study procedure and signed the consent form to participate.

A purposive sample of 32 patients (9–11 participants in each focus group) with OSAS using CPAP (37% female) in the age range of 37–77 years (*M* = 59.61; *SD* = 11.18) were enrolled in the study and participated in three focus groups.

Demographic and clinical information of the sample is reported in Table 1.

**Table 1:** Demographic and clinical information about the sample (*N* = 32).

	Focus Group 1 (*N* = 11)	Focus Group 2 (*N* = 11)	Focus Group 3 (*N* = 10)
**Gender**			
Male	6 (55%)	7 (64%)	3 (30%)
Female	5 (45%)	4 (36%)	7 (70%)
Age	*M* = 55.82; *SD* = 12.22; Range = 37–77	*M* = 61.91; *SD* = 9.52; Range = 43–76	*M* = 61.10; *SD* = 11.79; Range = 42–76
**Education level**			
High School	5 (45%)	6 (55%)	7 (70%)
Bachelor’s degree	4 (36%)	3 (27%)	3 (30%)
Master’s degree	2 (18%)	2 (18%)	0
Smoking			
smoker	4 (36%)	3 (27%)	3 (30%)
non-smoker	6 (55%)	5 (45%)	6 (60%)
ex-smoker	1 (9%)	3 (27%)	1 (10%)
**Living with…**			
Alone	7 (64%)	2 (18%)	3 (30%)
Partner	1 (9%)	7 (64%)	7 (70%)
Parent/parents	1 (9%)	0	0
Siblings	1 (9%)	0	0
Son/sons	1 (9%)	2 (18%)	0
Age at OSA Diagnosis, mean, range	45, 35–68	51, 45–70	47, 42–70
**Weight Status, *n* (%)**			
Underweight/normal weight (BMI ≤ 24.9 kg/m^2^)	0	0	0
Overweight (BMI 25.0–29.9 kg/m^2^)	0	0	0
Obesity Class I (BMI 30.0–34.9 kg/m^2^)	7 (64%)	8 (73%)	7 (70%)
Obesity Class II (BMI 35.0–39.9 kg/m^2^)	4 (36%)	3 (27%)	3 (30%)
Obesity Class III (BMI ≥40 kg/m^2^)			

### Procedure

Data were collected from August 2021 to January 2022, until data saturation was achieved. Data saturation was reached when no new themes emerged from additional participants (Morse et al., 2002; Silverman, 2010).

To minimize researcher bias, the interventions were led and moderated—using a phenomenological hermeneutic approach (Lindseth and Norberg, 2004)—by a female research psychologist (PhD) with no prior relationship with participants, who received specific training in conducting focus groups.

Individuals were made clear that their participation was voluntary, and that they could leave the focus group at any time—but none of them did. Participants were informed about the aim of the study and they knew that would participate in a face-to-face discussion with other patients with the same diagnosis and it would be the subject of scientific publication. Participants did not receive any reward/remuneration for participation in the study.

Focus groups were conducted during working hours and were held in a dedicated room of the hospital. No one other than the participants and the moderator was present in the room. All meetings were audio-recorded and lasted from 50 min to 1 h. To ensure consistency between groups, a semi-structured interview schedule guided the discussions (Table 2)—to investigate the lived experience participants made of OSAS diagnosis and CPAP treatment and to explore their general knowledge, attitudes, and beliefs about the disease and its treatment.

**Table 2:** Focus group questions.

What are the side effects of the treatment? What are the benefits you have experienced? Is it a problem to use CPAP? For whom? What was it that motivated you to start CPAP treatment? What motivates you to continue treatment? Were there any tiring moments when you thought about giving up using CPAP? What is the most tiring aspect of using CPAP? What problems have you experienced using CPAP? Did your partner help you in the decision to use CPAP? Has CPAP changed your quality of life? How? What expectations do you have about the treatment? Has having or not having a bed partner influenced your decision to use CPAP? How capable do you feel you are of managing CPAP? What or who might help you feel more capable? What things have helped you in using CPAP? Are there any aspects of treatment that you consider uncomfortable? How much would it help you to have a professional following you during use monitor and encourage you? What more can the CPAP provider do? What are the things you would tell a person who is about to start using CPAP? What are the recommendations? How satisfied are you with CPAP?

A storytelling approach with probing questions (such as “tell me more about that experience” and “how did that make you feel?”) was also used during the focus groups to clarify or expand the meanings presented by the participants, thus facilitating a dialogic interaction process. This is fundamental when conducting a phenomenological study to help participants express their deepest thoughts and feelings as freely as possible (Eatough and Smith, 2008). Each focus group ended when participants had nothing more to add. The interviewer did not field notes during or after the session.

Data collection also included age, sex, education, marital status, employment, and BMI—which were retrieved from patient medical records by a clinical psychologist working in the hospital and independent of the study. Data were registered and stored in a password-protected database, and only accessible by the clinician and the researchers.

### Data analysis and rigor

All recordings were transcribed verbatim and thematically coded by two researchers independently without using any software. Field notes were also taken for the examination of contextual information and general impressions during the sessions.

Transcriptions were analyzed qualitatively using the interpretative phenomenological analysis (IPA; Smith et al., 2009) to develop and label themes and sub-themes originating from the data.

In particular, IPA is most frequently applied to one-on-one interviews, and only a few studies have employed this approach to provide detailed examinations of personal lived experiences through focus group discussions (Palmer et al., 2010). Still, focus groups are an important source of experiential data (Palmer et al., 2010) as they (1) allow multiple voices to be heard in one sitting, thus drawing a larger sample into a smaller number of data collection events; (2) elicit more experiential reflection than one-to-one interviews; and (3) are cost-effective for hospital settings.

The data-driven approach (vs. theory-driven) was then used for text analysis, as considered the most effective way to investigate subjective experiences (Davidson et al., 2008). Comparison of findings with the research literature was carried out only at the end of the whole process as a sort of “return to the theory”—since emerging themes were derived inductively from the words of participants, rather than preconceived theoretical concepts and research evidence.

The analytical process initially involved line-by-line reading of each focus group transcript to provide a preliminary description of relevant topics, with notes recorded directly in the text. In this primary recursive phase, transcriptions were read and re-read, first for each session of the focus group, and then across sessions to let new insights and conceptual aspects dense with meaning come through. During the process, some themes were also dropped, for example, those that did not fit well with the emerging framework or those that were less represented.

Also, aspects within the themes that related specifically to the ongoing pulmonary rehabilitation program, and were therefore not transferable to a wider context, were not presented but fed back to the hospital as part of their evaluation process.

After the decoding procedure, disagreements between three coders were solved through extensive in-person discussion. Furthermore, the findings were shared and discussed with the multidisciplinary team, which improved the researchers’ reflexivity simultaneously reducing the influence of potential preconceptions and biases that could arise throughout the analytic process, with increased rigor.

The goal was to make sure that the analysis matched the participants’ accounts and that each account presented was justified by the data. Patients did not participate in the analytical process and did not provide their feedback on the findings.

## Results

### Living the diagnosis of OSAS as a shock

Some respondents experienced the diagnosis of OSAS as a traumatic event. This is particularly true in obese patients who attended the rehabilitation program with the aim of losing weight.

*“I found out about my apneas here* (during the weight loss rehabilitation program). *I have always slept, I did not realize I had this problem.”*

*“Doctors detected a high risk of apneas and I was shocked because I did not expect to have severe apneas with significant desaturations.”*

*“For me it was traumatic. I was 35 when they first told me I suffer from OSAS.”*

Often the diagnosis of OSAS comes as a surprise. Still, receiving a diagnosis of OSAS also surprised patients who are hospitalized for weight loss; it is an ominous diagnosis for patients, but also an explanation of the symptoms they have had for years whose symptoms were previously recognized and informed by significant others.

*“After the diagnosis, I told my mom that she was right, that I sometimes stopped breathing while sleeping. I have never believed but got mad at her because she used to wake me up.”*

The presence of significant others is very important in recognizing the prodromal signs of the disease, but often the person carrying the diagnosis is unaware that he or she has an airway problem while sleeping.

### You should not sleep on it: The importance of prevention

In addition to being poorly recognized and diagnosed, OSAS symptoms are often confused with other diseases, including mood disorders, and then treated with suboptimal solutions. This tendency significantly increases the likelihood of developing additional medical problems and compromising the quality of life of the person, thus driving the need for prompt informative and preventive actions. This was reflected in the narrations of the respondents.

*“All the mental confusion, the lack of memory that I could not explain, I thought I was depressed. There is not enough communication or explanation about this pathology, and you almost feel wrong.”*

*“Polysomnography could also be used as a routine screening test for the driving license. I was unaware of it* (OSAS) *until I started having overweight problems. People may be at risk when driving even if they do not know it because apnoea is a problem on which there is no communication and knowledge.”*

Patients agree that the diagnosis came late and that they had several other diagnoses. This lack of diagnosis pushed them toward doctor-shopping behaviors, to search for the cause of their symptoms. For this reason, sometimes patients did not feel heard and listened to by the health service. They agree that it is important to do more prevention and screenings at the doctor’s, especially to avoid risks when driving, as OSAS is responsible for many road accidents.

### The adjustment to CPAP

The patients adjusted to the device differently. Some of them fully accepted CPAP and experienced a process of real embodying process: the machine was considered an extension of themselves and part of their everyday life. These showed a positive attitude toward their use due to the perceived beneficial effects on their quality of life and health status.

*“I have been using it* (CPAP) *since 2018. It took a while to get used to it but now it is something I cannot do without because I use it even for a nap in the afternoon and I feel well”*

*“The CPAP makes me feel relaxed and breathe better. If I don’t have the CPAP with me - which rarely happens when I took short trips or when I cannot bring it with me - I have a lot of trouble falling asleep. It gives me such a sense of relaxation, and well-being that I have to use it.”*

As reported above, for some respondents, it was even difficult to think about living without the CPAP anymore. They were so used to it that they were not willing to give up. In addition, a portion of the patients also stated that the device acquired such a great value in their lives that they referred to it as if it was a person.

*“I welcomed it with open arms.”*

*“I use it since 2020, I had 95 apneas per hour and I no longer had the REM phase, I love my CPAP, nobody can take it away.”*

On the contrary, other respondents found it intolerable to be in the need of using the CPAP, and some of them even considered it a detrimental factor in their quality of life.

*“I don’t want to become addicted to it* (CPAP)*.”*

*“I’m now using it* (CPAP) *for 4-6 hours per night, but at first it was traumatic … After 3-4 hours of sleep, I just wanted to stop.”’*

They used the device, but for fewer hours than prescribed—although they recognized a slight improvement in their health status. These patients did not intimately consider CPAP as life-saving but as a weird, uncomfortable tool that they were forced to use.

*“We feel a little disadvantaged because, with this mask, we look like extraterrestrials …Pipes here, pipes there.”*

*“I have started to use it for one/two hours per night and now I use it a little longer, like four or five hours per night, but honestly, I’d rather be without it, I don’t like to use it. My aim in life is to stop using it, maybe by losing weight…I would be very happy. I will keep using it if I have to but it is not normal anyway. It’s a foreign body that I am forced to use and presses on my face.”*

Among the respondents, resistance to CPAP was not driven by health and medical concerns, but the feeling of annoyance, discomfort, and even hate. Their likelihood of adhering to the therapy was based on the hope of being able to do without. Therapy seemed to them a never-ending “battle*”*—as they did not want to surrender completely to the device.

*“Honestly, I would not use it anymore - and I will certainly do it sooner or later because, by losing weight, it can be possible. This is what I am here (at the hospital) for.”*

*“With CPAP, is a nonstop battle but I used it.’*

Moreover, not only is the CPAP a voluminous device but it also requires ongoing adjustments to perform adequately. Furthermore, patients struggle to accept the idea to be seen by other people wearing the mask, and for all these reasons they often chose not to take the device with them during work trips or holidays—meaning they do not see it as part of their routine and are not worried about not using it for days.

*“If I have to go to the mountains or the seaside for a weekend, I will not take it with me”*

*“I’ll go on holiday for ten days and share the room with other people and it is a little… it might bother them because obviously … and I feel a little embarrassed, that’s it.*

A third category of patients comprises those who have a more neutral approach to CPAP use: they rationally accepted the treatment due to its health benefit—without any particular emotional rebounds.

*“It’s like wearing glasses: if I can’t see anything, I get glasses; if I can’t breathe, I use CPAP. The benefits are more than the discomfort caused by the CPAP, trust me.”*

Balancing the pros and cons of the use of the CPAP, the health improvement appears to be more important than the discomfort caused by the device. The therapy is simply accepted and followed by the patient as the only solution to reduce the impact of their chronic condition.

### Barriers and facilitators to the use of CPAP

Adherence to therapy is fundamental to pursuing a healthy status—but for several reasons commonly difficult to achieve. In this regard, the respondents acknowledged both facilitator and barrier to adherence were acknowledged by the respondents.

In the first phase of the treatment—when patients receive the diagnosis and the process of acceptance and understanding of the disease begins—obtaining information on OSAS and CPAP, as well as being followed up by the medical team seemed to represent important facilitators for the use of the device.

*“I got used to CPAP while hospitalized - as they taught me how to manage it. A doctor came to check if everything was fine, to change the mask if needed… until I was ready to go home”*

*“Here (during hospitalization) I understood the importance of using CPAP. A month's stay here was very useful, as the doctors came to check on me during the night.”*

The presence of healthcare professionals helped the patients to worry less anxious and feel better, as they had the opportunity to ask questions, receive personalized treatment, and receive emotional support.

This was particularly relevant for “naïve” CPAP users since the device represents something very difficult to deal with from both a medical and a psychological point of view.

Hospitalization also offered the respondents the opportunity to meet other people suffering from the same conditions. This represented both a facilitator and a barrier to CPAP adherence. Indeed, when roommates had a positive attitude toward the CPAP and more experience with it, they could provide important practical support and technical advice to respondents, as well as emotional help.

*“My roommate was important to me because she helped me and she fixed my mask in the right position. She supported me a lot.”*

In contrast, roommates who showed a negative attitude toward CPAP fueled fears and doubts about therapy in the respondents.

*“In my room, there was a woman who used CPAP but she didn’t want it, she wanted to take it off because she felt more tired than rested. I don’t want to think I should use it, I wouldn’t use it (the CPAP)”*

The support from others was mentioned as a facilitator for the use of the CPAP even after the person returns home. In fact, the role of family members in helping patients to stay in therapy was fully recognized.

*“When I came home, my husband helped me, he used to tell me < wear the mask because you will feel better>, and he was happy because I was doing fine.”*

*“If there is no one who tells me to wear CPAP, I don’t wear it. Therefore, my granddaughter advised me <Grandpa every night I will come to check if you use it>.”*

The support received from their spouse or other family members was significant not only from a practical point of view (i.e., in wearing the mask or checking the machine) but also as emotional aid.

Accordingly, patients living alone felt less motivated to use the CPAP because of the absence of somebody encouraging and supporting them—including the medical team.

*“Since I live alone I stopped using it (the CPAP), and I started using it again here in the hospital two weeks ago.”*

*“…You can gain or lose weight and wonder if the machine is still well-calibrated. The happy/sad face* (on the device) *only shows if the mask is well placed but it does not tell you if the pressure is correct, so you don’t know if you need to contact the pulmonologist, the doctor, or the technician. Here* (in the hospital) *a pulmonologist comes to check if the machine is well-calibrated every morning and eventually fix it - but when you are at home it doesn’t happen.”*

*“I don’t know if something is wrong… doctors must tell me.”*

Relying on a support figure, especially during initial adjustment to the device, also represented an important emotional aid for the patients.

*“A support figure is useful because you suddenly find yourself with this mask without being mentally prepared…”*

*“Being followed up by somebody is important, it's like walking together with someone who gives you a hand, which makes you feel you are not alone. It's essential in my opinion.”*

Among the motivational factors, the interviewees also mentioned the digital solution (i.e., mobile apps) that informs patients about their signs of progress in the treatment of the disease and the correct use of CPAP.

*“I'm also fine with an app, telling me if everything is fine or if I need to call the technician for a check.”*

In addition to monitoring and providing feedback on CPAP usage, mobile applications help patients to develop self-management skills, which increase their self-efficacy and ability to deal with negative emotions associated with device use.

### Three in a bed

Sleep is often a shared experience with a partner for many adults. Since the hallmark symptoms of OSAS (i.e., snoring and apneas) occur during the night, partners are likely to experience sleep disturbance that may contribute to daytime exhaustion, problems at work, and in their intimate relationships—that might be overcome with the use of CPAP.

*“No one could stay with me because I snored so much, but with the mask, I have no problems anymore.”*

*“My wife is happy since I have been using CPAP because I don't snore anymore and we are sleeping together again.”*

In these quotes, patients reported that the quality of their partners’ sleep improved since they started to use the CPAP - and consequently, their couple relationship, as severe OSAS made in some cases necessary for them to sleep in separate rooms.

However, the presence of a “third” in a bed might also negatively impact couple intimacy—especially at CPAP initiation, with important implications for the patients’ likelihood to adhere to therapy.

*“It’s not the most sensual thing…”*

*“It’s like a Kamasutra between the pillow, the air hose, and the CPAP hose.”*

*“You might throw air on your partner's face, and it's embarrassing to wear the mask when you sleep with someone.”*

Patients noted that CPAP is a cumbersome device that negatively impacts libido as aesthetically unpleasing and not particularly appealing. They also reported feeling embarrassed about having to deal with the side effects of the mask while in bed: in some cases, partners may wear earplugs not to hear the noise of the machine, while others shelter themselves with pillows at night to avoid air blowing.

### The relationship with the healthcare system

The use of the CPAP implies for patients with OSAS to be in contact with the health care system, which provides them with the device and follow-ups at home or remotely for adaptation of some parameters. Still, patients often express concerns about the maintenance of the machine due to the not always responsive feedbacks from the healthcare system, and the long detailed procedure they need to follow.

*“One con is the supply of the CPAP spare parts, sometimes the healthcare system suffers delays.”*

*“A hot topic is communication with CPAP providers, the healthcare system and the motorization* (DMV), *as drivers’ license holders we are treated like drug addicts or alcohol abusers. I use the CPAP every night and the technician could download my data from the CPAP and report it directly to the DMV. Instead, I need to undergo a DMV test for no reason. Streamlining this procedure could be a benefit in my opinion.”*

Maintenance of CPAP has been shown to be a potential barrier for patients because it may require a great deal of time and resources and a constant relationship with the healthcare system and its bureaucracy. Sometimes, there are delays and problems in obtaining the CPAP and its spare parts. This could cause frustration in patients, and further put those less motivated at risk of not adhering to CPAP use.

Furthermore, patients recognized that having this disease involves dealing with DMV paperwork and doing periodic check-ups to get a driving license. This may represent a barrier, because the patient may neglect his disease, which may worsen, and prefer not to have these problems with the license.

The emerging themes and related illustrative quotes are reported in Table 3.

**Table 3:** Emerging themes and related illustrative quotes.

Themes	Quotations
1. Living the diagnosis as a shock	*“For me it was traumatic. I was 35 when they first told me I suffer from OSAS”*
2. You should not sleep on it: the importance of prevention	*“Polysomnography could also be used as a routine screening test for the driving license. I was unaware of it* (OSAS) *until I started experiencing overweight problems. People may be at risk when driving even if they do not know it because apnoea is a problem on which there is no communication and knowledge.”*
3. The adjustment to CPAP	Positive adjustment: “I welcomed it (the CPAP) with open arms.” Negative adjustment: “I do not want to become addicted to it (the CPAP).” Neutral adjustment: “It’s like wearing glasses: if I cannot see anything, I get glasses; if I cannot breathe, I use CPAP. The benefits are more than the discomfort caused by the CPAP, trust me.”
4. Barriers and facilitators to CPAP use	*“I got used to the CPAP while hospitalized - as they taught me how to manage it. A doctor came to check if everything was fine, to change the mask if needed… until I was ready to go home”*
5. Being three in a bed	*“You might shoot air on your partner’s face, and it’s embarrassing to wear the mask when you sleep with someone.”*
6. The relationship with the healthcare system	*“A hot topic is the communication with CPAP providers, the healthcare system, and the motorization* (DMV), *as those with driving licenses are treated like drug addicts or alcohol abusers. I use the CPAP every night, and the technician could download my data from the CPAP and report it to the DMV directly. Instead, I need to undergo a test at DMV for no reason, because the data can be reported directly. Streamlining this procedure could be a benefit in my opinion.”*

## Discussion

To our knowledge, this paper for the first time describes the lived experience that Italian inpatients with OSAS and comorbid obesity using the CPAP made of their pulmonary disease and its treatment.

During the focus group, most patients reported that the diagnosis of OSAS was experienced as a traumatic event because they were unaware of their symptoms, as also found in previous studies (Bakker et al., 2019; Weaver, 2019; Waldman et al., 2020).

Indeed, OSAS often goes undiagnosed because its symptoms are commonly mistaken for “normal” snoring, tiredness, or mood disorders—among others. Specifically, the multidirectional relationships between depression, disturbed sleep, and OSAS are a source of potential diagnostic confusion and may explain why OSAS and depression are both generally under-diagnosed among the general population (Schröder and O'Hara, 2005). Failure to recognize these conditions may lead to inappropriate treatment—with consequent worsening of the health status of the person. Recent studies demonstrate that CPAP therapy also contributes to reducing depressive symptoms in patients with OSAS (Garbarino et al., 2020). Accordingly, this content emerged from our focus groups, with one of the interviewees stating that he thought to suffer from depression instead of OSAS, and stressing the importance of informing preventive actions in sleep disorders.

Furthermore, several large epidemiological studies have shown a strong reciprocal association between weight gain and an increase in the odds of developing OSAS, to the point that the number of people with known sleep apnea continues to grow as the obesity epidemic worsens (Ong et al., 2013). While the treatment of chronic diseases is undeniably complicated by the presence of comorbidities, the results of this study show that the presence of obesity can represent an opportunity for the detection of OSAS symptoms in patients undergoing weight loss rehabilitation, thus preventing the worsening of snoring and OSAS, and the associated obesity-related comorbidities (i.e., hypertension, insulin resistance, and cardiovascular problems).

However, the use of the CPAP—the gold standard treatment for this chronic condition—may be frustrating and necessarily place persons with OSAS in the need to redefine their daily routine.

However, the process of adjustment to the device varied consistently among respondents depending on its perceived beneficial effects and the personal experience patients had with the device. Notably, resistance to CPAP was not influenced by the individuals’ medical condition or health concerns, but by the social and practical aspects surrounding its use: most respondents did not feel comfortable wearing the mask in the presence of other people or bringing it with them while traveling and, therefore, preferred to stop using the device for a few days regardless of the severity of their apneas.

Those who were more inclined to adhere to therapy were not naïve patients—probably because they already had the opportunity to experience the positive effects of the treatment. Among those most resistant to change, instead, the use of the device was frequently motivated by the hope of being able to do without—and since losing weight also reduces the symptoms of sleep apnea, the goal of living without CPAP seems to represent an important factor in facilitating weight loss among the respondents. These results confirm the importance of the constructs of motivation and self-efficacy in determining adherence to treatment referring to the Self-Determination Theory (Ryan and Deci, 2000). In fact, previous studies showed that patient education on the consequences of poor adherence is not sufficient, and in some cases, it is detrimental because it forced the decision to not adhere to the treatment. Conversely, as suggested by studies conducted with a motivational interviewing approach (e.g., Aloia et al., 2004; Sparrow et al., 2010; Dantas et al., 2015; see for a review: Rapelli et al., 2021a), working in line with the values and orientating them toward the health goal could be more effective.

Among acknowledged facilitators to CPAP use were being informed and followed up by the medical team, but also receiving support from both the professionals and other patients.

This is particularly true for patients at their first use of the device and this underlines the delicate phase of the first adjustment to the device and the importance played by social influence.

Moreover, as also demonstrated in previous studies (Hagedoorn et al., 2000; Rapelli et al., 2020, 2021b), receiving support (both emotional and in dealing with technical issues of the machine) from the partner leads to improved adherence and clinical outcomes in patients with OSAS using CPAP. On the contrary, adherence appears scant among patients living alone (Luyster et al., 2016; Rapelli et al., 2022). Indeed—as shown in other research (Sawyer et al., 2010)—unmarried patients in this study reported lower perceived ability to manage the device and fewer positive experiences with the CPAP compared to their married counterparts. Also, living alone may be a risk factor for late diagnosis of OSAS. This is particularly obvious when one of the most common presentations of OSAS is loud snoring bothersome to the bed partner.

Therefore, conceptualizing OSAS from a dyadic perspective—when possible—is likely to be more effective in identifying sleep problems and developing strategies to improve adherence to CPAP therapy than focusing solely on the individual (Ye et al., 2017). Indeed, studies show that CPAP might have an impact on co-sleeping (Lewis et al., 2004; Cartwright, 2008), and the quality of intimate relationships (Baron et al., 2009). Many stated their apprehension about sleeping all night with “wires” and a mask, while others were concerned about the effect that wearing the CPAP might have on their sleep partners and the couple functioning. The negative psychological effects of the equipment and harmful attitudes toward CPAP treatment identified as putative barriers to adherence to treatment mirror those found in previous studies (Broström et al., 2010; Shaw et al., 2012).

Another facilitator mentioned by inpatients is the support of an app/device that could be a source of motivation for the patient that could know signs of progress. The telemedicine is already considered a best practice in order to have positive health-outcomes related, and the literature demonstrated the increase in CPAP adherence levels among patients with OSAS who used mobile health devices (e.g., Isetta et al., 2017; Hu et al., 2021), but our study is the first that recognized the need of patients and the importance to actively engage the patient in the treatment.

Still, concerning barriers to CPAP adjustment, another emerging theme was the relationship with the healthcare system—whose long-term support was largely perceived as ineffective and not promptly available due to long bureaucratic procedures. This generates concerns in people, in particular among subjects suffering from OSAS and comorbid obesity, who wonder how the CPAP re-calibration will work in relation to their loss of weight.

One key limitation of this study relates to the inclusion of a convenient sample of patients with OSAS and comorbid obesity—with a consequent lack of generalizability of the results to the OSAS population. However, to yield insightful results, an important factor when selecting the sample is its homogeneity. Other possible limitations of this study, including not having distinguished between patients at their first use of the CPAP and those who already experienced CPAP treatment relapse(s); and not having specifically investigated the role of the presence of one or more comorbid conditions in influencing adherence to CPAP use among patients with OSAS. Giving voice to informal caregivers—and especially spouses—of this patient population would also have enriched the results of this study with additional reflections and suggestions for the implementation of care actions. The involvement of the patients’ sleeping partners and the exploration of their needs and psychological status are fundamental, as participants described the impact on the relationship as a potential barrier.

Furthermore, our findings further stress the importance of increasing patient awareness and knowledge to help their understanding of the health implications of OSAS, which can be done at the community level through public health awareness campaigns or during individual physician-patient encounters. Moreover, the implementation of customized motivational interventions would increase the inner reason to change and promote their self-efficacy, variables that are considered fundamental to improve adherence to treatment among patients with chronic diseases.

## Conclusion

The findings of this study reveal that, despite the fact that the decisions to use CPAP are individualized; the barriers to the adoption and possible adherence to sleep apnea treatment are a combination of functional and interpersonal concerns that depend largely on the patient’s support environment and early experiences and beliefs about the CPAP.

## Data availability statement

The raw data supporting the conclusions of this article will be made available by the authors, without undue reservation.

## Ethics statement

The studies involving human participants were reviewed and approved by Istituto Auxologico Italiano. The patients/participants provided their written informed consent to participate in this study.

## Author contributions

GR, LA, and GP contributed to the development of the study, analysis of the results, and writing of the manuscript. IB, IT, PF, and GC contributed to the development of the study and writing of the manuscript. All authors contributed to the article and approved the submitted version.

## Funding

This research was funded by the Italian Ministry of Health.

## Conflict of interest

The authors declare that the research was conducted in the absence of any commercial or financial relationships that could be construed as a potential conflict of interest.

## Publisher’s note

All claims expressed in this article are solely those of the authors and do not necessarily represent those of their affiliated organizations, or those of the publisher, the editors and the reviewers. Any product that may be evaluated in this article, or claim that may be made by its manufacturer, is not guaranteed or endorsed by the publisher.
