# Corrigendum: Assessing the needs and perspectives of patients with obesity and obstructive sleep apnea syndrome following continuous positive airway pressure therapy to inform health care practice: A focus group study

**DOI:** 10.3389/fpsyg.2023.1155578

**Published:** 2023-02-20

**Authors:** Giada Rapelli, Giada Pietrabissa, Licia Angeli, Ilaria Bastoni, Ilaria Tovaglieri, Paolo Fanari, Gianluca Castelnuovo

**Affiliations:** ^1^Psychology Research Laboratory, Istituto Auxologico Italiano IRCCS, Milan, Italy; ^2^Department of Psychology, Catholic University of the Sacred Heart, Milan, Italy; ^3^Pulmonary Rehabilitation Department, Istituto Auxologico Italiano IRCCS, Verbania, Italy

**Keywords:** obstructive sleep apnea syndrome, continuous positive airway pressure, obesity, focus group, interpretative phenomenological analysis, clinical psychology

In the published article, there was an error in the affiliations listed for author Giada Rapelli. Instead of affiliations 1, “Department of Psychology, Catholic University of the Sacred Heart, Milan, Milan, Italy” and 2, “Psychology Research Laboratory, Istituto Auxologico Italiano IRCCS, Milan, Lombardy, Italy” this author should just be affiliated to “Psychology Research Laboratory, Istituto Auxologico Italiano IRCCS, Milan, Italy.” The corrected affiliations are listed in this article.

The authors apologize for this error and state that this does not change the scientific conclusions of the article in any way. The original article has been updated.

